# Mannose-Containing Oligosaccharides of Non-Specific Human Secretory Immunoglobulin A Mediate Inhibition of *Vibrio cholerae* Biofilm Formation

**DOI:** 10.1371/journal.pone.0016847

**Published:** 2011-02-09

**Authors:** Ashlesh K. Murthy, Bharat K. R. Chaganty, Ty Troutman, M. Neal Guentzel, Jieh-Juen Yu, Syed Khalid Ali, Crystal M. Lauriano, James P. Chambers, Karl E. Klose, Bernard P. Arulanandam

**Affiliations:** South Texas Center for Emerging Infectious Diseases, Department of Biology, San Antonio, Texas, United States of America; Louisiana State University, United States of America

## Abstract

The role of antigen-specific secretory IgA (SIgA) has been studied extensively, whereas there is a limited body of evidence regarding the contribution of non-specific SIgA to innate immune defenses against invading pathogens. In this study, we evaluated the effects of non-specific SIgA against infection with *Vibrio cholerae* O139 strain MO10 and biofilm formation. Seven day old infant mice deficient in IgA (IgA^-/-^ mice) displayed significantly greater intestinal MO10 burden at 24 hr post-challenge when compared to IgA^+/+^ pups. Importantly, cross-fostering of IgA^-/-^ pups with IgA^+/+^ nursing dams reversed the greater susceptibility to MO10 infection, suggesting a role for non-specific SIgA in protection against the infection. Since biofilm formation is associated with virulence of MO10, we further examined the role of human non-specific SIgA on this virulence phenotype of the pathogen. Human non-specific SIgA, in a dose-dependent fashion, significantly reduced the biofilm formation by MO10 without affecting the viability of the bacterium. Such an inhibitory effect was not induced by human serum IgA, IgG, or IgM, suggesting a role for the oligosaccharide-rich secretory component (SC) of SIgA. This was supported by the demonstration that SIgA treated with endoglycosidase H, to cleave the high-mannose containing terminal chitobiose residues, did not induce a reduction in biofilm formation by MO10. Furthermore, the addition of free mannose *per se*, across a wide dose range, induced significant reduction in MO10 biofilm formation. Collectively, these results suggest that mannose containing oligosacchardies within human non-specific secretory IgA can alter important virulence phenotypes of *Vibrio cholerae* such as biofilm formation, without affecting viability of the microorganism. Such effects may contribute significantly to innate immune defenses against invading pathogens *in vivo* in the gastrointestinal tract.

## Introduction

The large surface area of mucosal membranes is a major portal of entry and site for colonization by microorganisms [1]. Mucosal surfaces are protected by fortified host defense mechanisms, including immunoglobulin A (IgA), the predominant immunoglobulin in these compartments [2]. The polymeric immunoglobulin receptor (pIgR) is expressed on the basolateral surface of epithelial cells and transports IgA as well as pentameric IgM from the lamina propria into mucosal secretions [3]. Upon pIgR-mediated IgA transcytosis, a portion of the pIgR is cleaved off and released as the secretory component (SC) bound to dimeric IgA, forming secretory IgA (SIgA) [4]–[7].

Secretory IgA has been shown to be involved in the clearance of immune complexes [8], extracellular neutralization of pathogen infectivity [9], and intracellular neutralization of bacterial lipopolysaccharide (LPS) and viruses within epithelial cells [10]. Much of the available evidence regarding the protective role of SIgA against pathogens is derived from studies of antigen-specific IgA [11]. However, there is an accumulating body of evidence to suggest that non-specific SIgA also may assist in mediating mucosal homeostasis and modulating inflammation at mucosal surfaces [12]. For example, we [13] have shown that mice deficient in pIgR displayed significantly enhanced intestinal inflammation in response to dextran sodium sulfate induced colitis. It is not clear how non-specific SIgA mediates these effects; however, several studies [14], including ours [13], point toward an important contribution of the secretory component (SC). More recent studies have shown the probable role of glycans on SIgA in mediating the recognition of bacterial polysaccharides in innate immune responses [15].

*Vibrio cholerae* is a Gram negative motile bacterium that is responsible for the life threatening exhaustive diarrheal disease, cholera [16]. Epidemics of cholera are observed in southern Asia, Africa, and South America and are still prevalent as seasonal outbreaks [16]. *V. cholerae* persists in the environment by forming biofilms [17] and in the human host, as suggested by the presence of biofilm-like bacterial aggregates present in stool samples from cholera-infected patients[16], [18]–[20]. Removal of such aggregates from stool-contaminated water has been correlated with significant reduction of infectivity [21]. Moreover, feces collected from cholera-infected patients also display *V. cholerae* coated with SIgA, suggesting an important contribution of IgA in interaction and elimination of the bacterium [18]. Given (a) the suggested role of non-specific SIgA in protection against mucosal pathogens, (b) the evidence of interaction between SIgA and *V. cholerae*, and (c) the correlation of biofilm formation with virulence, we evaluated the effects of SIgA on biofilm formation and virulence of *V. cholerae*.

In this study, we determined the effect of non-specific SIgA on the colonization and biofilm formation by O139 strain of *V. cholerae* (MO10) [22]. We found that infant suckling mice deficient in IgA displayed greater intestinal bacterial burdens than wild type pups following MO10 challenge. This effect could be reversed by feeding milk from wild type dams, suggesting a role for passively transferred non-specific SIgA in milk. Non-specific human SIgA, but not serum IgA or serum IgG and IgM, inhibited biofilm formation by MO10 *in vitro*, without affecting viability of the bacterium. The inhibitory effect on biofilms was reversed by removal of the high mannose containing oligosaccharides from SIgA, or could be induced by free mannose, suggesting an important role for oligosaccharides within SIgA in mediating this effect.

## Results

### Non-specific SIgA is involved in reduction of *Vibrio cholerae* colonization *in vivo*

**Figure 1:**
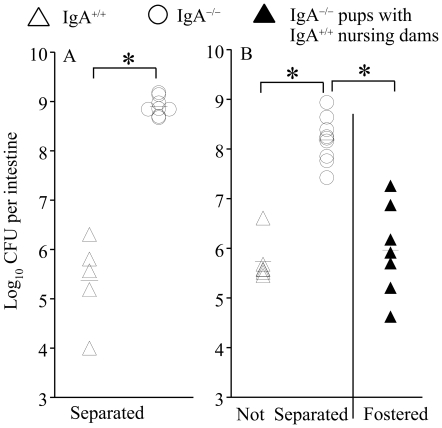
Significance of IgA in inhibition of colonization. Groups (*n* = 5–7) of seven-day old infant suckling BL6/129 (IgA^+/+^) and IgA^-/-^ pups were challenged intra-gastrically through the oral route with 10^6^ CFU of MO10. The mice were either (A) separated from the nursing dams or (B) housed with the nursing dams of the same strain, or IgA^-/-^ pups were fostered with IgA^+/+^ nursing dams. At 24 hr after challenge, small intestines were removed, homogenized, and the bacterial burdens analyzed. Each marker represents an individual pup and the mean±SD also is shown. * Significant difference in numbers of bacteria observed between the indicated groups (*p*≤0.05, Student's *t* test). Results are representative of two to three independent experiments.

We evaluated whether SIgA could alter the phenotype of *V. cholerae* infection *in vivo* using an established model of oral intragastric *V. cholerae* challenge in infant suckling mice [23]. Groups of 7-day-old infant suckling IgA^-/-^ and IgA^+/+^ mice, either separated (Fig 1A) or not separated from their mothers, or IgA^-/-^ pups cross-fostered with lactating IgA^+/+^ mothers (Fig 1B) were challenged with *V. cholerae*. To evaluate the effects of innate non-specific IgA, the bacterial burden in the small intestines was measured at 24 hr post-challenge. As shown in Fig 1A, IgA^-/-^ infant suckling mice separated from their mothers displayed significantly (*p*≤0.05) enhanced intestinal bacterial burden (>3 log_10_) when compared to the IgA^+/+^ pups. Additionally, IgA^-/-^ infant suckling mice not separated from their mothers displayed significantly (*p*≤0.05) greater intestinal bacterial burden (>2 log_10_) when compared to the IgA^+/+^ pups (Fig 1B), suggesting the importance of IgA in controlling intestinal *Vibrio* infection. Importantly, IgA^-/-^ suckling infant mice cross-fostered with lactating IgA^+/+^ mothers displayed a significant (*p*≤0.05) reduction (∼2 log_10_), in intestinal bacterial burden when compared to IgA^-/-^ pups with IgA^-/-^ dams (Fig 1B), suggesting the contribution of non-*Vibrio* specific SIgA in milk to the control of intestinal *V. cholerae* infection.

### Non-specific human SIgA inhibits biofilm formation by *Vibrio cholerae*

**Figure 2:**
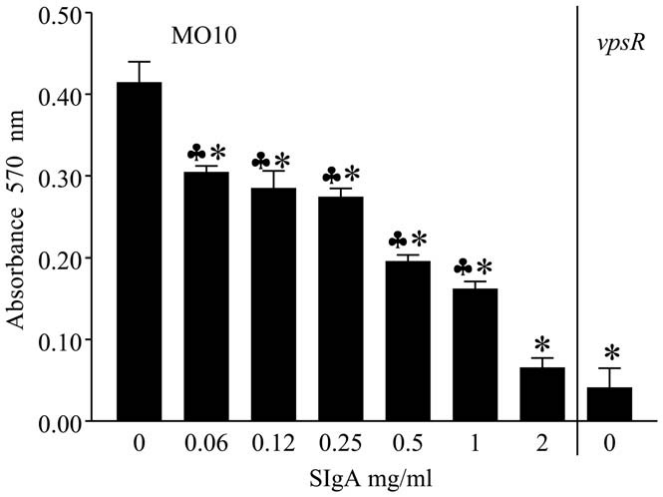
Dose dependant effect of non-specific human SIgA on MO10 biofilm formation. Overnight cultures of MO10 were diluted and incubated without shaking for 24 hr at 30° C with escalating doses of human non-specific SIgA. MO10 without SIgA and the biofilm mutant *vpsR* were evaluated as positive and negative controls, respectively. Biofilm formatin was measured using crystal violet staining. Significant (*p*≤0.05, Student's *t* test) difference in biofilm formation between * indicated group and PBS, and indicated group and *vpsR*. Results are representative of three independent experiments.

Based on *in vivo* evidence in the infant mouse model that non-specific SIgA contributes to the reduction of virulence and/or colonization of *V. cholerae*, and that biofilm formation is associated with virulence of this bacterium [24], we further investigated the role of human non-specific SIgA on biofilm formation by *V. cholerae*. Overnight cultures of *V. cholerae* 0139 (MO10) were diluted to approximately 2×10^6^ CFU/ml in 2X LB, and 50 µl was incubated at 30°C for 24 hr with escalating doses of human non-specific SIgA in 50 µl PBS or PBS alone. Wells with the *V. cholerae* mutant (*vpsR*) that is deficient in biofilm production were evaluated as negative controls. The biofilm formation was measured as described previously [25], [26]. As shown in Fig. 2, wild type MO10 displayed significantly (*p*≤0.05) enhanced biofilm formation (0.41±0.03) compared to *vpsR* (0.04±0.02). The addition of SIgA to MO10 cultures significantly (*p*≤0.05) reducted biofilm formation across the entire examined dose range (0.06 to 2 mg/ml). At the highest dose examined (2 mg/ml), the biofilm formation by MO10 was comparable to that induced by *vpsR*, a mutant deficient for biofilm formation. As expected, culture wells with PBS or LB broth (no bacteria) did not display biofilm formation.

**Figure 3:**
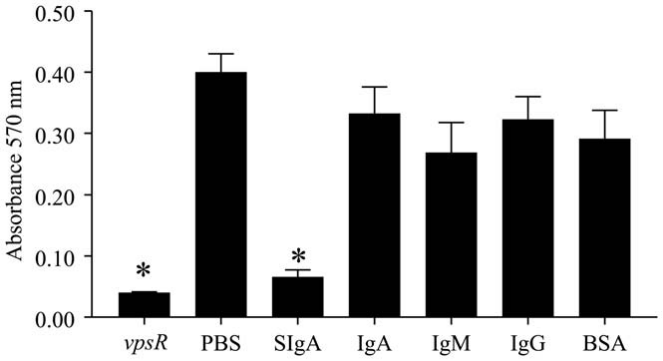
Influence of non-specific SIgA and serum human immunoglobulins on MO10 biofilm formation. Overnight cultures of MO10 were diluted and incubated without shaking for 24 hr at 30° C with 2 mg/ml doses of human non-specific SIgA or serum IgA, IgG, IgM, or BSA. MO10 without immunoglobulin and the biofilm mutant *vpsR* were evaluated as positive and negative controls, respectively. Significant (*p*≤0.05, ANOVA) difference in biofilm formation between * indicated group and PBS. Results are representative of three independent experiments.

**Figure 4:**
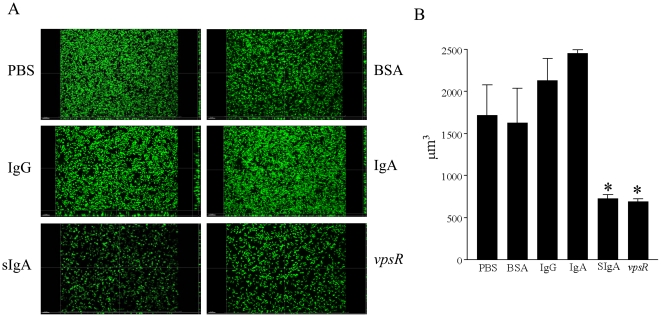
Quantification of inhibiton of MO10 biofilm formation by non-specific human SIgA. Overnight cultures of GFP-expressing MO10 were diluted and incubated without shaking for 24 hr at 30° C with a 2 mg/ml dose of human non-specific SIgA or serum IgA, IgG, IgM, or with BSA. MO10 without immunoglobulin and the GFP expressing biofilm mutant *vpsR* were evaluated as positive and negative controls, respectively. (A) Three dimensional images were obtained using a Zeiss Axiovert 200M microscope equipped with an apotome. (B) The respective isosurface volumes were quantified using Imaris software. Significant (*p*≤0.05, ANOVA) difference in biofilm formation between * indicated group and *V. cholerae* grown in the presence of serum IgG or serum IgA. Results are representative of three independent experiments.

Since non-specific SIgA inhibited biofilm formation by MO10, we evaluated whether serum immunoglobulins (IgA or IgG or IgM) were capable of inducing similar effects using the highest examined dose of 2 mg/ml for each immunoglobulin. As shown in Fig 3, incubation of *V. cholerae* MO10 induced a high level of biofilm formation (0.41±0.02) and as expected, the *V. cholerae vpsR* mutant displayed minimal biofilm (0.04±0.001) formation. Importantly, biofilm formation by *V. cholerae* MO10 in the presence of SIgA (0.07±0.01), but not serum IgA (0.33±0.04), IgM (0.27±0.05), IgG (0.32±0.04), or an unrelated antigen BSA (0.29±0.05), was significantly (*p*≤0.05) reduced when compared to PBS (0.41±0.02). These results suggest that human non-specific SIgA, but not serum IgA or other immunoglobulin isotypes, plays an important role in inhibiting biofilm formation by *V. cholerae*. Additionally, the effects of SIgA and serum immunoglobulins on biofilm formation were quantified by measuring isosurface volume. As shown in Fig 4, the isosurface volume of biofilm by *V. cholerae* MO10 upon incubation with SIgA was reduced compared to incubation with serum IgA, IgG, IgM, control BSA or PBS alone. As expected, the *vpsR* mutant displayed a low isosurface volume of biofilm. We also quantified the biofilm isosurface volume in these different treatment groups to confirm the qualitative results. As shown in Fig. 4B, MO10 grown with added PBS displayed a high isosurface volume (1723±353 µm^3^) of biofilm, which was comparable to that induced in wells that included BSA (1632±403 µm^3^), serum IgA (2133±261 µm^3^), or IgG (2455±43 µm^3^). However, the isosurface volume of biofilm was significantly (*p*≤0.05) reduced in wells containing MO10 incubated in the presence of SIgA (728±42 µm^3^), or in wells of the biofilm mutant *vpsR* (690±35 µm^3^). In fact, the addition of SIgA to MO10 reduced the biofilm isosurface volume to levels comparable to that of the biofilm deficient *vpsR*.

We further determined whether the reduction in biofilm formation of MO10 was due to bacteristatic or bactericidal effects induced by SIgA. Cultures of MO10 were grown for 24 hr in the presence of SIgA, serum IgA, serum IgG, or BSA. None of the immunoglobulins, including SIgA, displayed any significant change in growth characteristics of MO10 when compared to BSA (data not shown). Collectively, these results demonstrate the efficacy of non-specific SIgA in inhibiting the biofilm formation without affecting viability of *Vibrio cholerae*.

### Mannose containing oligosaccharides of non-specific human SIgA mediate inhibition of MO10 biofilm formation

**Figure 5:**
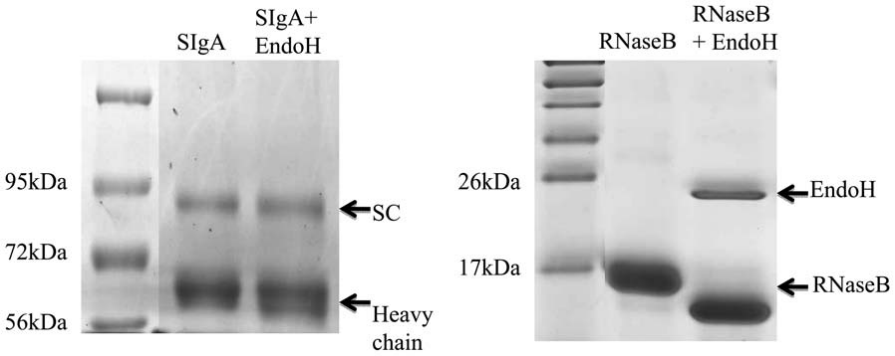
Digestion of SIgA with EndoH. 2 mg/ml of SIgA alone (lane 1) or SIgA with EndoH in buffer (lane 2), RNaseB alone (lane 3) or RNaseB with EndoH (lane 4) were incubated for 4 hr at 37°C, and then subjected to SDS-PAGE and stained with Coomassie blue. Results are representative of three independent experiments.

**Figure 6:**
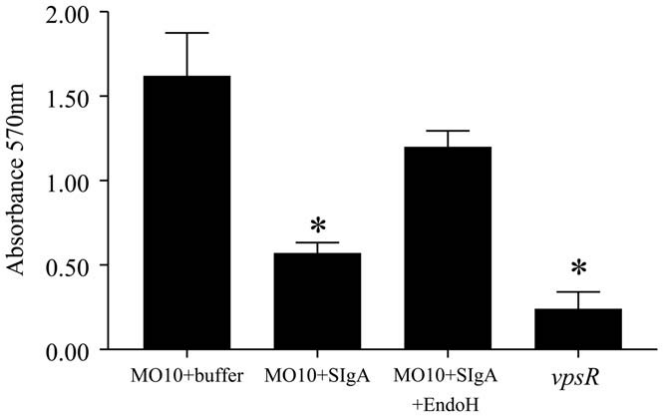
Inhibition of MO10 biofilm formation through high mannose residues on oligosaccharide side chains of non-specific human SIgA. Overnight cultures of MO10 were diluted and grown with 2 mg/ml of SIgA, or SIgA treated with EndoH, or without immunoglobulin for 24 hr at 30°C. Biofilm formation was quantified as before using crystal violet stain. Significant (*p*≤0.05, ANOVA) difference in biofilm formation between * indicated group and MO10 without SIgA. Results are representative of three independent experiments.

**Figure 7:**
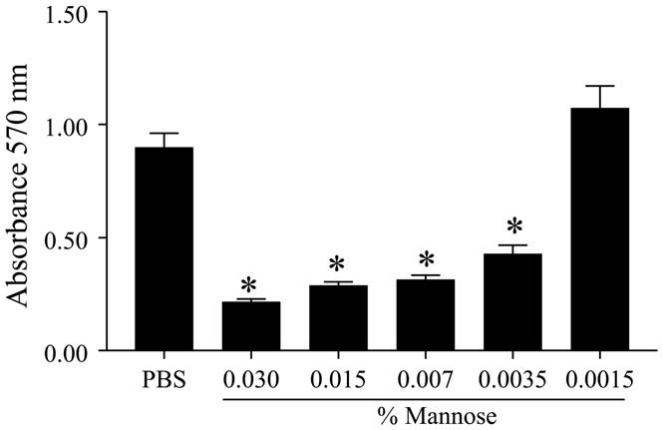
Inhibition of MO10 biofilm formation by free mannose. Overnight cultures of MO10 were diluted and grown with progressively reducing doses of mannose, or without mannose (PBS) for 24 hr at 30°C. Biofilm formation was quantified as before using crystal violet stain. Significant (*p*≤0.05, ANOVA) difference in biofilm formation between * indicated group and MO10 without mannose. Results are representative of three independent experiments.

Since SIgA, but not other serum immunoglobulins, inhibited biofilm formation by MO10, we hypothesized that the high oligosaccharide content in the secretory component of SIgA [27] may be responsible for the observed effects. To address this possibility, SIgA was treated with the enzyme EndoH to cleave the chitobiose core and release the high mannose terminal sugar residues. As shown in Fig. 5, EndoH-treated SIgA displays a reduced molecular weight of the SC and IgA heavy chain when compared to untreated SIgA, suggesting removal of oligosaccaharides. Since the change in the molecular weight of SIgA was low, we also incubated EndoH with a positive RNaseB control and found clear reduction in the molecular weight of RNaseB, suggesting the efficient glycosidase activity of EndoH. Additionally, human non-specific SIgA (200 µg) was incubated with EndoH (25,000 units) in sodium citrate buffer (G5 buffer) at 37°C for 4 hr. The remaining EndoH was removed by filtration and the EndoH-treated SIgA was incubated with overnight *V. cholerae* culture, diluted to 2×10^6^ CFU/ml, in equal amounts at 30°C without shaking. Untreated SIgA in buffer and buffer alone were used as positive and negative controls, respectively, for inhibition of biofilm formation by MO10. As shown in Fig 6, MO10 expectedly produced significantly (*p*≤0.05) greater biofilm formation (1.62±0.25) compared to vpsR (0.24±0.1). The addition of untreated SIgA (0.57±0.076), but not EndoH-treated SIgA (1.2±0.1), induced a significant (*p*≤0.05) reduction in biofilm formation by MO10. To confirm our hypothesis further, we evaluated whether addition of free mannose could inhibit biofilm formation by MO10. Cultures of MO10 were compared for biofilm formation with addition of increasing concentrations (0.0125% to 0.1%) of free mannose in PBS. As shown in Fig 7, mannose significantly (*p*≤0.05) inhibited biofilm formation by MO10 in a dose dependent fashion (0.22±0.01 at 0.03%; 0.28±0.01 at 0.015%, 0.31±0.02 at 0.007%; 0.43±0.04 at 0.0035%) when compared to cultures with PBS added alone (0.9±0.06), and the inhibitory effect of mannose was lost with very low doses of mannose (1.1±0.1 at 0.0015%). Together, these results suggest that the terminal high-mannose residues on the SIgA contribute significantly to the inhibition of biofilm formation by *V. cholerae*.

## Discussion

Secretory IgA is the principal immunoglobulin at mucosal sites and plays an important role in extracellular/intracellular neutralization and immune exclusion, functions that maintain mucosal homeostasis [12]. While the role of antigen-specific SIgA has been well studied in various models of infectious disease [11], the contribution and function of non-specific SIgA in modulating innate defenses is underappreciated. In this study, we demonstrate that non specific SIgA plays an important role in control of intestinal *Vibrio cholerae* infection. Moreover, non-specific human SIgA, but not serum IgA, IgG and IgM, was found to inhibit biofilm formation by *V. cholerae*, in a process that was mediated by the high mannose containing oligosaccharides of SIgA.

Antigen-specific immune responses including Ag-specific SIgA have been shown to be involved in protection against pathogens. In general, SIgA has been shown to neutralize pathogen infectivity in the mucosal lumen, during transcytosis in intraepithelial cell compartments, and in the lamina propria [8]–[10]. Specifically with regard to *V. cholerae*, Guentzel et al. [28] demonstrated that the anti-flagellar sheath SIgA and SIgA directed against naked flagella or outer membrane microvesicles from non-flagellated organisms reduced the virulence of the pathogen in the suckling infant mouse model [29]. Since motility was associated with virulence of *V. cholerae* in the infant mouse model [30], a suggested mechanism was that the organisms were prevented from traversing the mucus layer thus gaining access through intervillous spaces down to the intestinal crypts; these relatively “unstirred” regions were observed to be a primary niche of the bacterium during infection [28], [31]. Recent reports also support these observations by demonstrating that mucosal immunization with outer membrane vesicles induced anti-*V. cholerae* LPS antibodies that inhibit bacterial motility and reduce the virulence of *V. cholerae* in the infant suckling mouse model [32]. There was a minimal role for bacterial killing and/or anti-cholera toxin antibodies in these observed effects in both the early and recent studies. Collectively, antigen-specific SIgA clearly plays a role in defense against *V. cholerae*.

In this study, we focused on the contribution of non antigen-specific SIgA against *V. cholerae* infections. A role for the free secretory component (SC) has been suggested in innate immunity against various pathogens including enterotoxigenic [33] and enteropathogenic *Escherichia coli* [34], indirectly implicating a role for the SC of SIgA in the observed effects. Specifically, these studies employed *in vitro* model systems and demonstrated a dose dependent effect of milk components including free SC on pathogen-binding and/or blocking pathogen infectivity. It also was shown that glycans in free SC are responsible for binding and reducing the infectivity of enteropathogenic *E. coli* and the cytopathic affect of *Clostridium difficile* toxin A [35]. We extend these observations by demonstrating that SC-containing SIgA, but not non-SC containing immunoglobulins including serum IgA, IgG and IgM, are capable of inhibiting biofilm formation by the MO10 strain of *V. cholerae*. This suggested that the SC itself of SIgA may be responsible for the biofilm inhibitory effects. This effect of SIgA was dose dependent and was observed even at 0.06 mg/ml, a concentration below the physiological levels present in mucosal secretions. The biofilm inhibitory effect of SIgA was dependent upon the oligosaccharides, since cleaving the chitobiose core reversed the effect. Additionally, the terminal residues of the oligosaccharide side chains contain high-mannose residues, and the addition of free mannose inhibited biofilm formation by MO10, collectively suggesting that the terminal high mannose residues in oligosaccharides in SIgA may be predominantly responsible for inhibition of biofilm formation by *Vibrio cholerae*.

It is to be noted that the biofilm inhibitory properties of SIgA did not involve bacterial killing. Similar results were reported recently by Bishop *et al.* [32] that the anti-*V. cholerae* LPS antibody reduces bacterial motility and infectious burden independent of bacterial killing. Additionally, Guentzel *et al* [28] demonstrated that treatment with anti-IgA reversed the immobilization of motile vibrios allowing them to breach the mucus barrier and spread along the villi, and was associated with reversal of protection in animals immunized with flagellar or vesicular vaccines. The reduction of infectious burden without an effort to eliminate the pathogen appears to align well with the hypothesis that mucosal immunoglobulins protect without eliciting much inflammation [36], given the expected serious consequences of strong inflammatory responses at mucosal surfaces. This also is supported by the finding that IgA, as opposed to the predominant serum immunoglobulin IgG or IgM, does not activate the classical complement pathway, and is at best a weak inducer of the alternative complement pathway [37], [38]. Thus, it appears that the mucosal immunoglobulin IgA evolved to meet the tailored needs for protection of mucosal surfaces [36]. To this end, it has been demonstrated recently that most, if not all, jawed vertebrate taxa display a functionally similar but specific mucosal immunoglobulin isotype IgA (mammals and birds), IgX (amphibians), or IgT (bony fish) [39]. IgA, IgX, and IgT have been suggested to be products of convergent evolution, implying the recruitment of a unique immunoglobulin to mucosal immunity at least three times [39]. Collectively, these results emphasize the unique nature of SIgA when compared to other immunoglobulins in host defenses against pathogens.

Biofilm formation has been suggested to be an important aspect of virulence in *V. cholerae* [17]. Specifically, removal of *V. cholerae* biofilm-like aggregates from cholera stools reduces the infectivity significantly [21]. Therefore, SIgA and/or free SC may act as host defense mechanisms to curtail the formation of such biofilm-like aggregates *in vivo* and reduce the virulence of *V. cholerae*, an idea clearly supported by the results from our *in vivo* experiments. IgA^-/-^ infant suckling mice displayed significantly enhanced intestinal *V. cholerae* burden as early as 24 hr post challenge compared to IgA^+/+^ pups, suggesting a role for innate, non antigen-specific, mechanisms in the protective effect, as also has been shown previously [40]. In fact, this study by Hsiao *et al.*, 2006 also demonstrated that the mannose sensitive hemagglutinin (mshA) of *V. cholerae* is important for binding to SIgA, and that this mannose sensitive interaction prevents the bacterium from penetrating mucus barriers and attaching to epithelial cells. The results of the current study confirm and extend these observations by demonstrating that intestinal colonization by *V. cholerae* and *in vitro* biofilm formation are reduced upon interaction with SIgA in a mannose dependent fashion. Such protective effects of IgA were apparent both in IgA^+/+^ pups separated from the IgA^+/+^ dams, or if the dams were allowed to nurse the pups. Importantly, the reduction in bacterial burdens with the cross-fostering of IgA^-/-^ pups with IgA^+/+^ mothers suggests that passive transfer of IgA in milk contributed to the protective effect. In mucosal secretions including milk, IgA is secreted as dimeric SIgA, thus suggesting that non-specific SIgA contributes to the reduction in *V. cholerae* burden in the intestines. Additionally, the enhancement of intestinal bacterial burden in IgA^-/-^ suckling infant mice occurred despite the presence of an intact polymeric immunoglobulin receptor (pIgR), and therefore presumably normal levels of free SC, suggesting that the SIgA containing SC plays a predominant role in innate protective immunity against *V. cholerae in vivo*. This is supported by reports that differences in oligosaccharide composition between serum IgA and SIgA may also result from differences in IgA1 and IgA2 composition, which differ substantially in oligosaccharide content [5]. Specifically, serum IgA is primarily composed of IgA1, whereas SIgA is an approximately equal mixture of IgA1 and IgA2, and IgA2 contains greater oligosaccharides than IgA1 [5]. However, a role for SC is supported by other studies which show that the SC in human milk binds to *Clostridium difficile* toxin A receptors [41], and thus may inhibit toxin-mediated disease, suggesting that differences in oligosaccharide composition within IgA subclasses itself, or due to the presence of SC may contribute to protection against mucosal pathogens. The implications of such effects may be manifold including, but not limited to, (a) reduction in intestinal pathogenic burden, (b) reduced concentrations of toxins in the gut microenvironment, and/or (c) reduced systemic spread of the pathogen, all subsequently resulting in reduced disease manifestation.

In summary, this study demonstrates that high mannose containing oligosaccharides of SIgA play an important role in reducing the intestinal burden of *Vibrio cholerae*. Beyond the well characterized adaptive immune defenses by antibodies, these results suggest the involvement of mucosal immunoglobulins in innate host defenses against mucosal pathogens in general. Given that primary IgA deficiency affects approximately 1 in 500 people [42], the results of this study underscore the need to further evaluate in detail the nature of the predisposition of these individuals to gut pathogens.

## Materials and Methods

### Ethics Statement

All animal experiments were performed in compliance with the Animal Welfare Act, the U.S. Public Health Service Policy on Humane Care and Use of Laboratory Animals and the “Guide for the Care and Use of Laboratory Animals” published by the National Research Council. The University of Texas at San Antonio Institutional Animal Care and Use Committee (IACUC) approved this study under the protocol number MU013-08/11A0.

### Bacterial strains

A virulent *Vibrio cholerae* O139 strain, MO10 [22], was used for this study and a defined mutant of MO10 *flaA*::Cm _*vpsR* _*lacZ* designated *vpsR* [26], defective in biofilm formation, was used as a control for the experiments. For isosurface volume quantification, green fluorescent protein (GFP) expressing strains of MO10 and *vpsR* were utilized.

### Infant mouse colonization assays

Mice were housed and bred at the University of Texas at San Antonio and provided food and water *ad libitum*. Animal care and experimental procedures were performed in compliance with the Institutional Animal Care and Use Committee (IACUC) guidelines. Groups (*n* = 5-7) of 7-day old IgA^+/+^ (BL6/129) and IgA^-/-^ mice [43] were challenged through the oral route intragastrically with 10^6^ CFU of MO10 in sterile saline using intramedic tubing. In one experiment, pups were separated from the mothers in the group. In the other, the pups were allowed to remain in the cage with nursing dams. Additionally, one group of IgA^-/-^ pups were fostered with IgA^+/+^ nursing dams as a source of breast milk SIgA. Approximately 24 hrs later, pups were euthanized and the entire small intestine was removed. Small intestines were homogenized and serial dilutions plated on LB agar containing 50 µg/ml streptomycin (Sigma Aldrich, St. Louis, MO) and incubated for 24 hr at 37°C for determination of *Vibrio cholerae* burdens.

### Biofilm formation

A micotiter plate assay was adapted for the evaluation of biofilm formation. Briefly, *V. cholerae* 0139 (MO10) and the *vspR* mutant were grown overnight at 37° C with shaking in LB containing 50 µg/ml streptomycin. The bacterial cultures were diluted in 2X LB to a concentration of approximately 2×10^6^ CFU/ml and 50 µL was inoculated into sterile 96-well polystyrene microtiter plates along with 50 µL of PBS, or bovine serum albumin (BSA) (Fisher Scientific, Fairlawn, NJ) or serum immunoglobulin A (IgA, Pierce, Rockford, IL) or secretory IgA (SIgA) (Sigma Aldrich) or human serum IgG (Pierce) or human serum IgM (Pierce) at a final concentration of 200 µg/well in PBS. In another experiment, escalating doses of free mannose (Difco, Detroit, MI) (serially doubling from 0.0015% to 0.03%) were added to cultures of MO10. Cultures were incubated without shaking for 24 hr at 30° C, removed, and the plates washed 3X with PBS and allowed to dry. Biofilms were quantified using crystal violet staining for 15 min as described previously [25]; [26], washed with 1X PBS, and the absorbance was measured at 570 nm using a µQuant ELISA plate reader (BioTek, Winooski, VT).

### Three-dimensional imaging

MO10 was grown overnight at 37° C in LB containing 50 µg/ml streptomycin. Overnight cultures were diluted to a final concentration of approximately 2×10^6^ CFU/ml using fresh LB with streptomycin and kanamycin and 150 µL of this suspension was inoculated into sterile 24-well polystyrene microtiter plates containing cover slips. Proteins were added to the wells containing MO10 at a concentration of 2 mg/ml in a total volume of 150 µL and the cultures were allowed to incubate for 24 hr at 30° C without shaking. One set of triplicate wells containing the vpsR mutant, defective for biofilm formation, was evaluated as a negative control. After incubation, the cover slips were removed and washed with sterile 1X PBS, and mounted on glass slides with FluorSave Reagent (Calbiochem, La Jolla, CA). Slides were imaged using a Zeiss Axiovert 200M (Carl Zeiss Vision Microimaging Inc., Thornwood, NY) microscope equipped with an apotome and analysis of volume was performed using Imaris software (Bitplane Inc., Saint Paul, MN).

### High mannose digestion assay and biofilm formation

High mannose sugars on SIgA were enzymatically removed using Endoglycosidase H (Endo H, New England Biolabs, MA) digestion. Briefly, 200 µg of SIgA was incubated in the presence of EndoH (25,000 units) and G5 buffer at 37° C for 4 hr. A positive RNaseB control provided by the manufacturer also was used to evaluate the glycosidase activity of EndoH. Following the 4 hr incubation period, sample mixtures were subjected to SDS-PAGE electrophoresis to confirm the glycosidase activity of EndoH. Following EndoH treatment, SIgA also was loaded on a Microcentricon (MilliPore, MA) of 100 kDa cutoff to separate the cleaved sugars. The treated SIgA was resuspended in 50 µL of sterile PBS and incubated with MO10 for biofilm formation as described earlier.

### Statistical Analyses

SigmaStat (Systat Software Inc., San Jose, CA) was used to perform tests of significance. The Student's *t* test was used to compare two groups. For comparison between multiple groups, one-way analysis of variance (ANOVA) was used. All experiments were repeated and analyzed independently.
